# Organizing phenotypic data—a semantic data model for anatomy

**DOI:** 10.1186/s13326-019-0204-6

**Published:** 2019-06-20

**Authors:** Lars Vogt

**Affiliations:** Institut für Evolutionsbiologie und Ökologie, Rheinische Friedrich-Wilhelms-Universität Bonn, An der Immenburg 1, 53121 Bonn, Germany

**Keywords:** Phenotypic data, Semantic data model for anatomy, Instance anatomy knowledge graph, Anatomy, ontology, Zoology, Knowledge management, Morphological description, Morphology

## Abstract

**Background:**

Currently, almost all morphological data are published as unstructured free text descriptions. This not only brings about terminological problems regarding semantic transparency, which hampers their re-use by non-experts, but the data cannot be parsed by computers either, which in turn hampers their integration across many fields in the life sciences, including genomics, systems biology, development, medicine, evolution, ecology, and systematics. With an ever-increasing amount of available ontologies and the development of adequate semantic technology, however, a solution to this problem becomes available. Instead of free text descriptions, morphological data can be recorded, stored, and communicated through the Web in the form of highly formalized and structured directed graphs (semantic graphs) that use ontology terms and URIs as terminology.

**Results:**

After introducing an instance-based approach of recording morphological descriptions as semantic graphs (i.e., *Semantic Instance Anatomy Knowledge Graphs*) and discussing accompanying metadata graphs, I propose a general scheme of how to efficiently organize the resulting graphs in a tuple store framework based on instances of defined named graph ontology classes. The use of such named graph resources allows meaningful fragmentation of the data, which in turn enables subsequent specification of all kinds of data views for managing and accessing morphological data.

**Conclusions:**

Morphological data that comply with the here proposed semantic data model will not only be computer-parsable but also re-usable by non-experts and could be better integrated with other sources of data in the life sciences. This would allow morphology as a discipline to further participate in eScience and Big Data.

**Electronic supplementary material:**

The online version of this article (10.1186/s13326-019-0204-6) contains supplementary material, which is available to authorized users.

## Background

Morphological data drives much of the research in the life sciences [1]. Unfortunately, however, the morphological record consists for the most part of unstructured text. This has far-reaching consequences for research based on morphological data, since conventional morphological free text descriptions not only bear problems relating to terminology and lack of semantic transparency, but they also cannot be parsed by computers. Someone interested in using morphological data, for instance, for systematically searching for correlations between phenotypic data and genotypes over a broad set of non-model organisms, will soon be discouraged after briefly having delved into the relevant morphological literature, having not only to search for relevant data in published morphological descriptions, but also having to deal with morphological terms, the meaning of which depends on the described taxon, the describing author and the time when the description has been conducted. Moreover, while some terms refer to common spatio-structural properties, others refer to a common function or a presumed common evolutionary origin, or some mixture of those three categories (see *Linguistic Problem of Morphology* [2]). As a consequence, interpreting and analyzing morphological data becomes unnecessarily difficult for non-experts and integrating morphological data with other sources of data in the life sciences very difficult and time-consuming.

This is unfortunate because morphology offers a treasure trove of valuable data that only has to be harvested. Morphological data remain the primary data source for defining most species and understanding their phylogenetic history but are also important for recognizing, defining, and diagnosing pathological conditions in plants, animals, and other organisms [1]. Morphological data also provide insights to questions of development, function, evolution, and interaction of phenotypes with their environments, yielding in the discovery of new pharmaceutical agents and the development of new materials and technical solutions. Therefore, if morphological data would be easily accessible, re-usable by non-experts, and computer-parsable, they could significantly contribute to a better understanding in a diverse field of research areas within the life sciences, ranging from evolution and ecology to pharmacy, health, engineering, and material sciences. In an ideal world, this data would be openly accessible and easily findable through the Web, they would be stored in online databases in a highly formalized and structured syntax and format, they would be semantically transparent and thus easier to comprehend and interpret, and data from different authors and different taxa could be easily integrated and algorithms could read and analyze them.

Since we are living in the age of Big Data, Linked-Open-Data, and the Semantic Web, this ideal world should not be too far-off for morphology. Efficiently managing and organizing data has become key to data exploration and eScience, and developing adequate data and metadata standards is becoming increasingly important [3–5]. eScience is a new driving force for scientific progress in data-rich fields of empirical research [6] and requires data and metadata to be maximally findable, accessible, interoperable, and reusable (FAIR guiding principle [7]). This can be achieved by establishing semantic transparency and making data and metadata computer-parsable [8–10]. Ontologies and other controlled vocabularies have taken a central role in this context because they provide the required standardized semantic structure for data and metadata to become comparable and computer-parsable (e.g., [11–13]).

Ontologies are vocabularies that are used for describing a certain reality. They consist of terms with commonly accepted definitions that are formulated in a highly formalized canonical syntax and standardized format, such as the Web Ontology Language (OWL) serialized to the Resource Description Framework (RDF), with the goal to yield a lexical or taxonomic framework for knowledge representation [14].

Unfortunately, in morphology, the use of ontologies is still very limited. Currently, only a small fraction of morphological data is eScience-compliant and that fraction is usually restricted to data about model organisms (e.g., [15–19]). However, an increasing amount of taxon-specific ontologies is becoming available (e.g., Hymenoptera Anatomy Ontology [20, 21]; Spider Ontology [22]; Plant Ontology [23]; Vertebrate Trait Ontology [24]; Uberon multi-species anatomy ontology [25]; Cell Ontology [26]). A first step towards increasing the amount of eScience-compliant morphological data is to semantically enrich free text descriptions with ontology terms. This is a step in the right direction. However, semantic technology would be used to its full potential only when the entire description is represented using Uniform Resource Identifiers (URIs) with the RDF’s syntax of *Subject*, *Predicate*, and *Object* and when storing these triples as semantic graphs in a tuple store. A semantic graph is a network of RDF/OWL-based triple statements, in which some URIs take the *Object* positions in some triples and the *Subject* position in other triples, connecting several triples to form a semantic graph.

Tuple stores are capable of handling large semantic graphs, and semantic technology facilitates detailed data retrieval of RDF/OWL-based data through SPARQL endpoints [27], as well as inferencing over OWL-based data through semantic reasoners [28]. Some tuple stores such as the Jena tuple store framework [29] allow for organizing the store into different physically separate RDF stores, with each RDF store being structured into different named graphs. A named graph identifies a set of triple statements by adding the URI of the named graph to each triple belonging to this named graph, thus turning these triples into quads. The Jena tuple store framework can handle such quads. The use of named graphs thus enables partitioning data in an RDF store and enables making statements about statements comparable to OWL reification, but outperforms the latter for more complex queries [30].

In the last decade, progress has been made in semantically representing phenotypic data. Two alternative basic approaches have been suggested for documenting the anatomical organization of a given specimen using ontology terms:

### Class-based approach

A particular morphological phenotype is described by defining an ontology class according to a set of properties that are characteristic to that phenotype. In other words, the definition of the ontology class contains the description of the phenotype. The description itself is thus contained in class axioms, which in OWL are expressed as TBox expressions. The morphological description of a specimen that bears the phenotype then merely expresses that this specimen instantiates the respective ontology class. Corresponding descriptions have been called ***Semantic Phenotypes***. In this class-based approach, *Semantic Phenotypes* are therefore defined through TBox expressions that are formally described following an Entity-Quality (EQ) scheme [31–34].

### Instance-based approach

A particular morphological phenotype is described by generating a semantic graph consisting of instances that instantiate ontology classes, with each instance referring to a particular part of the structure to be described. The corresponding descriptions are thus contained in ABox expressions and are called ***Semantic Instance Anatomy Knowledge Graphs*** (or *Semantic Instance Anatomies* [2, 12, 35–37]; from here on *Anatomy Knowledge Graphs*). Contrary to the *Semantic Phenotypes* approach, the *Anatomy Knowledge Graphs* approach follows a more modular framework that makes use of anatomical entity terms from existing ontologies and does not necessarily require the definition of new ontology classes to represent a given phenotype. Besides the smaller demand for additional ontology classes, *Anatomy Knowledge Graphs*, when compared to *Semantic Phenotypes*, have usually better properties regarding retrievability of data, for instance, when attempting to retrieve the numbers out of measurement data. The reason for that is that querying TBox expressions is more difficult than querying ABox expressions. When querying TBox expressions, the basic graph-pattern-matching of SPARQL has to be defined using entailment regimes [38], which is more complex and computationally difficult under full expressivity of OWL [39, 40]. Therefore, querying *Anatomy Knowledge Graphs* is more straightforward and computationally less difficult than querying *Semantic Phenotypes*. Moreover, the instance-based approach allows for identifying and re-using every individual part and property that the description mentions because each of them possesses its own URI. As a consequence, *Anatomy Knowledge Graphs* can be easily fragmented into sub-graphs. This is not possible when applying the class-based approach, because its TBox expressions treat all described parts and properties as anonymous resources (for a detailed comparison of the *Semantic Phenotypes* approach and the *Anatomy Knowledge Graphs* approach see [41]).

In the following, I give a brief introduction to *Anatomy Knowledge Graphs* and their accompanying metadata before I will propose a general scheme of how to efficiently organize respective morphological data and metadata in a tuple store.

## Methods

### Organizing a document as a semantic graph

In RDF, propositions are structured as triple statements consisting of *Subject*, *Predicate,* and *Object*, with *Subject* and *Predicate* being resources in the form of a Uniform Resource Identifier (URI) and the *Object*, depending on the type of *Predicate* used in the statement, being either a resource or some numerical or literal value. A resource always refers to a real thing or a piece of data (e.g., a Web page), and the value can be a unique ID, a numerical value, an arbitrary label, a proper name, a kind name, or a string of free text. An RDF statement can be modeled as a graph, with *Subject* and *Object* forming nodes that are connected through a *Predicate* as a labeled directed arch (edge). Since a given URI can take the *Object* position in one statement and the *Subject* position in another statement, triple statements can connect to each other and jointly form a semantic graph. Such semantic graphs can be used for representing specific properties of a particular anatomical entity or its relation to other anatomical entities of a given specimen and thus for describing the anatomical organization of that specimen.

**Fig. 1:**
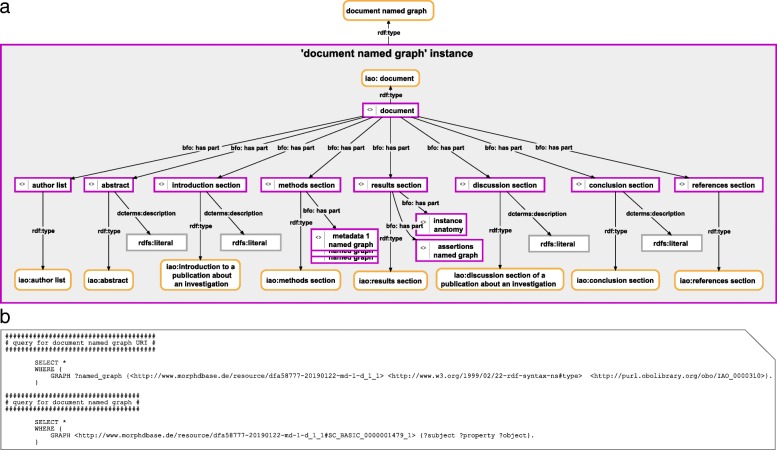
Instance-based semantic graph of a document and its parts with a corresponding SPARQL Query. **a** An instance-based semantic graph representing the structure of a document, stored in a named graph instance of the ontology class ‘document named graph’. The graph relates an instance of the IAO class ‘document’ to instances of other IAO classes, all of which refer to different parts of a typical scientific publication. Some of these parts may themselves have parts, which are modeled as a semantic graph (here not shown), whereas others only have a literal value associated. *Purple-bordered box = ontology individual; purple-bordered grey box = named graph instance; yellow-bordered box with rounded corners = ontology class; grey-bordered box = literal or numerical value; arrow = property*. **b** A two-step SPARQL Query to access the named graph shown in A. The first query searches for the URI of the named graph. We know the URI of the entry, which is the instance of the IAO class ‘document’, and we know that the document named graph contains the triple statement *Subject*: ‘document’, *Predicate*: ‘rdf:type’, *Object*: ‘iao:document’. We thus search for the named graph that contains this triple statement. In the second query we retrieve all triple statements contained in this named graph (the result of the second query is documented in Additional file 1)

However, simply storing a large semantic description graph in a database and making it openly accessible is usually not an efficient way of organizing morphological data and does not comply with the FAIR guiding principles. Additional information must be provided when being published in a database. One way would be to treat a semantic morphological description as a scientific publication, and thus organize it according to the commonly used general structure for scientific publications. This would require modeling semantic morphological descriptions as documents that consist of several different parts, including abstract, author list, introduction, methods, results, discussion, conclusion, and references section. The description itself would be part of the results section of the document. Some of these sections may contain information that is usually not easily modeled in a semantic graph and may instead be recorded as a string of free text (which, in turn, could be enriched with semantic annotations). The Information Artifact Ontology (IAO [42]) provides an adequate model for structuring the corresponding document into these parts and the corresponding semantic graph could be stored in an instance of a corresponding ontology class ‘document named graph’ (Fig. 1). Respective instance-based semantic document graphs can be stored in tuple stores and made accessible for machine-readable service requests through a SPARQL endpoint, whereas a human-readable version must be accessible for browser requests (for an example see Additional file 1).

### Morphological descriptions as Semantic Instance Anatomy Knowledge Graphs

When describing the anatomical organization of a given specimen using instances of ontology classes, the resulting semantic graph represents the anatomy of an individual organism and thus a *Semantic Instance Anatomy*. Instantiated anatomy is the field of anatomy that comprises anatomical data pertaining to individual organisms and their parts, as opposed to canonical anatomy, which is the field of anatomy that comprises the synthesis of generalizations based on anatomical observations describing idealized anatomy [43].

**Fig. 2:**
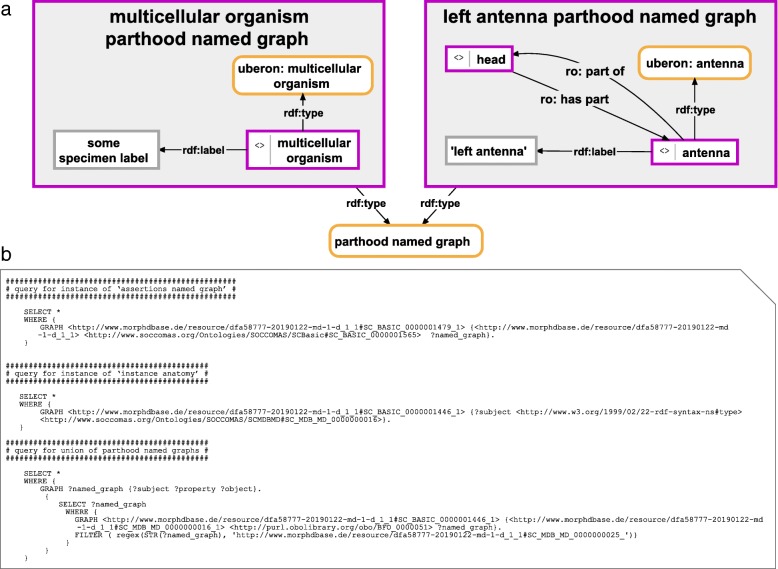
Instance-based semantic graphs of parthood relations with a corresponding SPARQL Query. **a** Left: shows the parthood named graph instance associated with the anatomical entity that is at the root of the partonomy. It is an instance of the ontology class ‘parthood named graph’ and contains the URI of the root entity (i.e., the entity that is being described) together with its class affiliation and its human readable label. **a** Right: shows the parthood named graph instance associated with some parts of the root entity. It is also an instance of the class ‘parthood named graph’, but contains, in addition to its own URI, class affiliation and human readable label, also the URI of its direct parent entity and the parthood relation between them. The union of all instances of ‘parthood named graph’ of a given *Semantic Instance Anatomy* documents the direct parthood relations of all described parts together with their class affiliation and their human readable label. For reasons of clarity, resources are not represented with their URIs but with labels. *Purple-bordered box = ontology individual; purple-bordered grey box = named graph instance; yellow-bordered box with rounded corners = ontology class; grey-bordered box = literal or numerical value; arrow = property*. **b** A three-step SPARQL Query that makes a union of all parthood named graphs of a given document. The first query searches for the URI of the instance of ‘assertions named graph’ in the ‘document named graph’. The second query searches for the instance of ‘instance anatomy’ within this assertions named graph. The sub-query of the third query searches within this assertions named graph for all triple statements that have *Subject*: ‘instance anatomy’ and *Predicate*: ‘bfo:has part’ and retrieves a list of named graph resources. The result of this sub-query is filtered for individual named graph resources of the type ‘parthood named graph’. The main query provides the union data of the filtered named graphs from the sub-query (the result of this query is documented in Additional file 2)

**Fig. 3:**
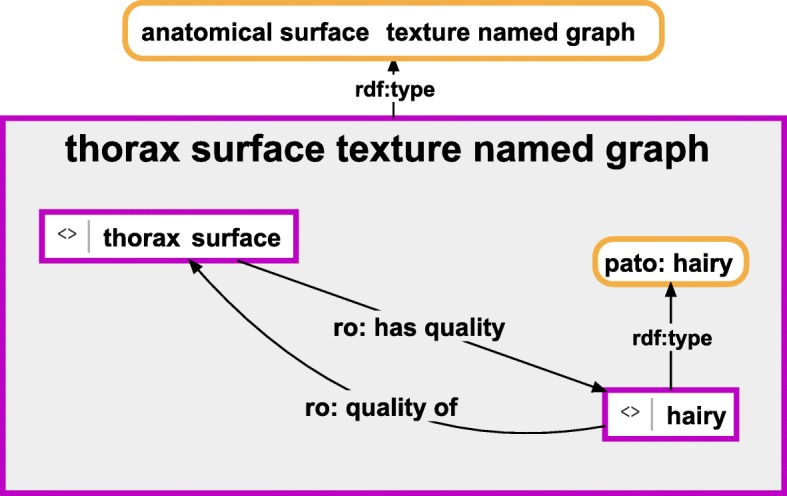
Instance-based semantic graph of a quality specification of an anatomical entity**.** An instance-based semantic graph documenting the quality of a particular thorax surface, stored in a named graph instance of the ontology class ‘anatomical surface texture named graph’. The graph relates the instance of an anatomical surface with an instance of a subclass of the class ‘texture’ of PATO together with the class affiliation of the latter. For reasons of clarity, resources are not represented with their URIs but with labels. *Purple-bordered box = ontology individual; purple-bordered grey box = named graph instance; yellow-bordered box with rounded corners = ontology class; arrow = property*

**Fig. 4:**
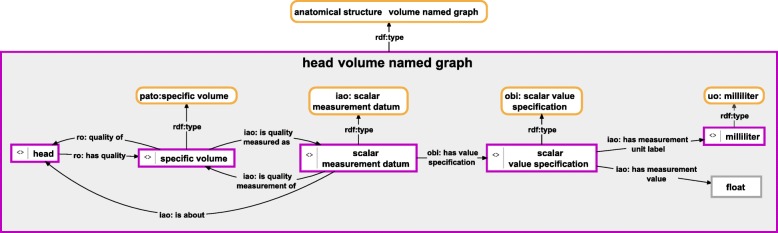
Instance-based semantic graph of a measurement value of a particular quality of an anatomical entity**.** An instance-based semantic graph documenting the volume measurement of a particular head, stored in a named graph instance of the ontology class ‘anatomical structure volume named graph’. The graph relates an instance of ‘head’ of the Hymenoptera Anatomy Ontology with an instance of ‘specific volume’ of PATO together with the measured value in the form of a float input and an instance of ‘milliliter’ of the Units Ontology specifying the measurement unit. For reasons of clarity, resources are not represented with their URIs but with labels (Additional file 4 documents the SPARQL Query for this named graph together with its result). *purple-bordered box = ontology individual; purple-bordered grey box = named graph instance; yellow-bordered box with rounded corners = ontology class; arrow = property*

Due to the fact that *Semantic Instance Anatomy Knowledge Graphs* (short: *Anatomy Knowledge Graphs*) are instance-based semantic graphs consisting of sets of ABox expressions, every individual part and property mentioned in an *Anatomy Knowledge Graph* possesses its own URI and we can fragment the graph into several flexibly manageable but yet meaningful sub-graphs without losing any information. By storing all triple statements that correspond to a single descriptive statement in their own named graph, we utilize this fragmentability of *Anatomy Knowledge Graphs*. A single descriptive statement could, for instance, refer to a particular parthood relation between two anatomical entities, to the texture inhering in a particular anatomical surface, or to the volume measurement of a particular anatomical structure (Figs. 2, 3, 4). *Anatomy Knowledge Graphs* can be organized into several such sub-graphs, with each named graph instance having its own URI. Named graphs of the same type can be organized within an ontology as instantiating the same class of named graph. The class-based semantic graphs of *Semantic Phenotypes* cannot be fragmented this way because all individual parts and properties mentioned in the corresponding defining TBox expressions are anonymous resources and cannot be reorganized in several sub-graphs without losing information.

Class-subclass relations provide the hierarchical backbone across the various classes of an ontology. In an *Anatomy Knowledge Graph*, in contrast, the corresponding backbone is provided by the parthood relations across the various instances of anatomical entities mentioned in the description. Since each instance resource mentioned in an *Anatomy Knowledge Graph* instantiates an ontology class of an existing ontology, applications that process and analyze *Anatomy Knowledge Graphs* can utilize both types of hierarchies, i.e., taxonomies and partonomies, for inferencing and for quantitatively comparing different *Anatomy Knowledge Graphs* [35–37].

Following the idea of fragmenting the description into single descriptive statements, each statement about a particular parthood relation between two anatomical entities can be stored in a named graph instance of a ‘parthood named graph’ ontology class. Except for the anatomical entity that is placed at the root of the partonomy, each parthood statement consists of an anatomical entity *X* and an anatomical entity *Y*, with *Y* being a direct part of *X*, and the parthood relation between them. The graph also specifies the class membership of entity *Y* and its human-readable label. The anatomical entity at the root of the partonomy has its own special semantic graph for its parthood named graph, which only specifies its class membership and its human-readable label (see Fig. 2). When forming the union of all the instances of ‘parthood named graph’ belonging to the same *Anatomy Knowledge Graph*, one receives a semantic graph that specifies the direct parthood relations between the described anatomical entities together with their class affiliation and their human-readable labels (see Additional file 2).

This partonomy backbone can be expanded to include further propositions that describe each entity of the partonomy in more detail. Each such proposition will result in a corresponding semantic graph with its own specific structure, which must be stored and organized in a named graph resource that belongs to a specific named graph class. We can, for instance, distinguish different categories of qualities of a part such as its shape, color, texture, etc. Not all categories may be applicable to all parts. However, each category has its own named graph class assigned (see Fig. 3). Moreover, we can document measurement values associated with specific qualities of a part and distinguish measured qualities by using different named graph classes (e.g. Fig. 4).

Each named graph class has its own associated data standard, i.e., its own data scheme for the semantic graph it contains. Moreover, all semantic graphs that document measurement values must follow the same general scheme of semantically representing measurement values wherever applicable. When rigorously applied, the set of different named graph classes with their associated data schemes provide an eScience-compliant standard for morphological data that guarantees the comparability of semantic graphs belonging to different *Anatomy Knowledge Graphs*. Each named graph class can thereby be understood to correlate with a specific perceptual question that can only be answered by studying the specimen. That question functions like a perceptual category that is part of a general morphological structure concept [2, 8, 12].

### Metadata relevant to morphological descriptions

Morphological descriptions are often based on evidence from more than one individual specimen. This is especially the case when the description covers macroscopically visible anatomical entities as well as anatomical entities that are only visible using a specific type of microscope. Many imaging methods used in morphology require not only the use of a specific microscope but also a detailed and very specific preparation and processing of the specimen prior to its microscopic examination. These preparation and processing steps are usually irreversible, with the consequence that different imaging methods cannot be used with the same individual specimen as their common source of evidence. Moreover, different imaging methods are not only applied to enable studying anatomical entities at different levels of resolution but are in general applied for visualizing different aspects of a specimen’s anatomical organization that are otherwise not directly visible to the naked eye. Different preparation methods aim at visualizing different anatomical aspects, and often only the combination of different imaging methods allows reliable identification of the existence and the location of a specific type of anatomical entity within an organism.

As a consequence, detailed morphological descriptions that cover different aspects of the anatomical organization of an organism, different levels of granularity, and the identification of the existence and location of different types of anatomical entities are usually based on a set of several individual specimens, with each single specimen being specifically prepared and processed prior to its examination. Therefore, if we want to document all metadata that are relevant to a particular morphological description, we must consider that different parts of the description will refer to different specimens and thus have different sets of metadata associated with it.

Documenting this kind of differentiated metadata is common practice for morphological descriptions published as unstructured free text in conventional journals. It is not common practice, however, for documenting metadata associated with *Semantic Phenotypes* because it is technically challenging to assign different metadata sets to different parts of the phenotype description contained in a *Semantic Phenotype* due to the fact that the description takes the form of TBox expressions. The here proposed *Anatomy Knowledge Graph* approach, in contrast, allows for such differentiated metadata documentation. Each single metadata set can be documented in a separate semantic graph, which at any time can be integrated with the semantic description graph.

Metadata directly relevant to morphological descriptions refer to the actual observation process and all contextual information relevant to the observation-based statements that the morphological description contains. Any such observation process relates to a particular processed specimen as its input and one or more descriptive statements (i.e., morphological data) as its output. It thus represents a planned process that results in information that is based on the examination of a given particular material entity.

**Fig. 5:**
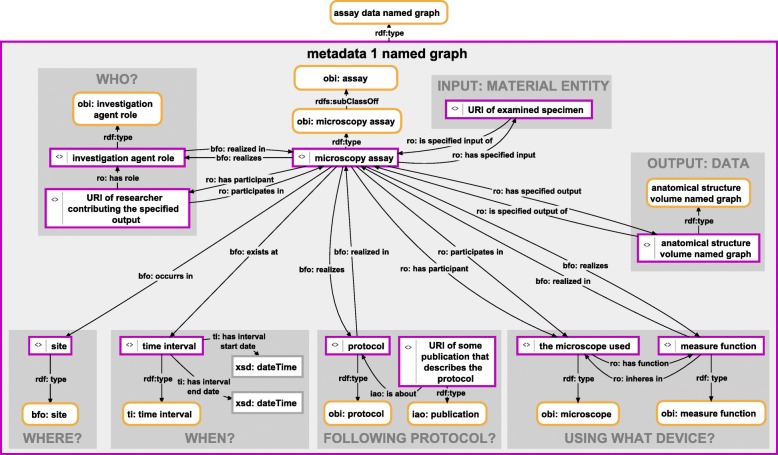
Instance-based semantic graph of a morphological observation process using a microscope**.** An instance-based semantic graph documenting a morphological observation process using a microscope, stored in a named graph instance of the ontology class ‘assay data named graph’. The graph relates an instance of ‘microscopy assay’ of IAO with instances of various other ontology classes from OBI and IAO, specifying who conducted the observation, where and when it took place, following which protocol and using which device (i.e., microscope). The semantic graph furthermore specifies the particular material entity that served as subject and thus as input of the observation process (i.e., the particular specimen), and it specifies the data that is the output of the observation, which is contained in one or more named graph instances, each being a part of an *Anatomy Knowledge Graph*. For reasons of clarity, resources are not represented with their URIs but with labels. *Purple-bordered box = ontology individual; purple-bordered grey box = named graph instance; yellow-bordered box with rounded corners = ontology class; arrow = property*

Analogous to the *Anatomy Knowledge Graphs* discussed above, in order to be maximally comparable and to comply with the FAIR guiding principle, semantic graphs documenting observation processes should be stored in instances of the same corresponding named graph class and should comply with the same underlying general data scheme. The Ontology for Biomedical Investigations (OBI [44]) provides an adequate scheme that can be applied for documenting such metadata as a semantic graph. An instance of the class ‘obi:assay’ (OBI_0000070) takes a central position within a graph that documents a particular observation process. This ‘obi:assay’ instance is linked to the processed specimen, which is specified as its input. It is also linked to the named graph instance that contains that part of the *Anatomy Knowledge Graph* that documents the output of this particular observation (Fig. 5). In this way, we can make metadata statements about a particular observation (which, in turn, is contained in its own named graph; see above). This linking between metadata sets and particular observations is possible because the latter are organized in their own particular named graph and each named graph is a resource itself, and thus can be used in triple statements as an *Object* or a *Subject*. If the different metadata sets and the different particular observations of a given description were not organized in their own named graphs, we could not individually link them and would thus lose important information, for example, on which of several specimens a given observation was based. In the class-based *Semantic Phenotypes* approach, different metadata sets cannot be assigned to different parts of the description of a phenotype because this description is contained in TBox expressions within the class axioms, with all mentioned parts and properties being anonymous, i.e., without their own URI.

Further information can be linked to the ‘obi:assay’ instance, such as the devices that have been used (e.g., the microscope), following which protocol, who conducted the observation, and where and when [44, 45] (Fig. 5). Specific substances that have been used during the assay and that must be documented as well can be added to the graph as additional input.

**Fig. 6:**
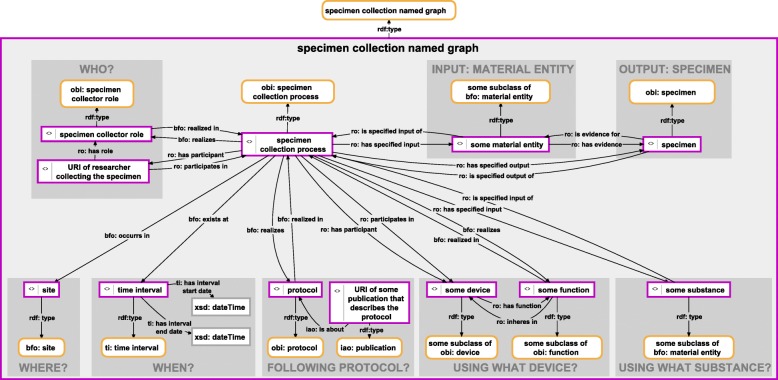
Instance-based semantic graph of a specimen collection process. An instance-based semantic graph documenting a specimen collection process, stored in a named graph instance of the ontology class ‘specimen collection named graph’. The graph relates an instance of ‘specimen collection process’ of OBI with instances of various other ontology classes from OBI, IAO and other ontologies, specifying who conducted the observation, where and when it took place, following which protocol and using which device (e.g., a live trap). The semantic graph furthermore specifies the particular material entity that served as subject and thus as input of the collection process (i.e., the particular material entity), and it specifies the specimen that is the output of the collection process. For reasons of clarity, resources are not represented with their URIs but with labels. *Purple-bordered box = ontology individual; purple-bordered grey box = named graph instance; yellow-bordered box with rounded corners = ontology class; arrow = property*

Besides documenting all information relating to the observation process itself, morphological descriptions usually have additional relevant metadata. These metadata relate to the studied specimen, its corresponding collection metadata, and its particular history of collection, identification, and preparation processes. Especially all processes-related specimen data may be documented in a separate specimen document or require additional named graph classes that refer to the different types of processes that have to be documented, thereby following established standards for documenting specimen-related data [44, 46–49] (for an example of a specimen collection semantic graph see Fig. 6).

**Fig. 7:**
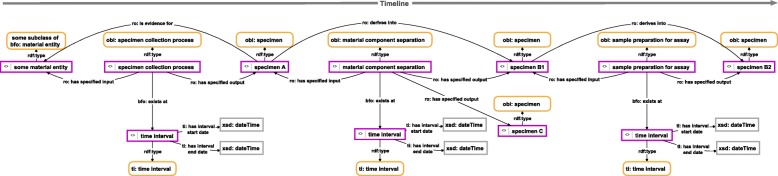
Instance-based semantic graph of a specimen history. The graph documents the history of all specimens derived from an initially collected specimen (instance A). These specimens resulted from various processes of material processing, in which some specimen served as input and the changed specimen as output. For reasons of clarity, resources are not represented with their URIs but with labels. *Purple-bordered box = ontology individual; yellow-bordered box with rounded corners = ontology class; arrow = property*

Since each process of material processing changes the input material (i.e., the particular specimen), we are often dealing with a chain of specimen resources that relate to one another through time. And in case the input material is separated into different parts, as for instance when extracting a DNA sample or some tissue sample from an existing specimen, the process may even result in two separate output materials, in which case the initial chain forks into two separate chains. Such a specimen-history can be documented in a semantic graph as well (see example in Fig. 7).

## Results

**Fig. 8:**
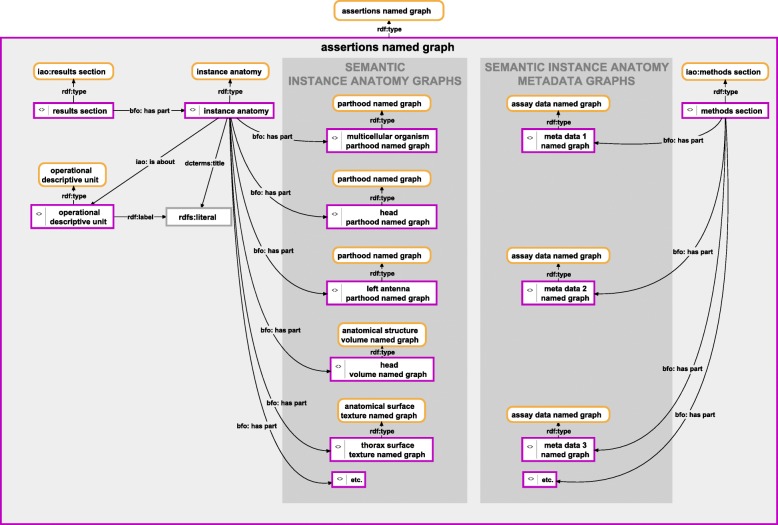
Instance-based semantic graph of the overall assertions. An instance-based semantic graph documenting the overall assertions relating to a morphological description, stored in a named graph instance of the ontology class ‘assertions named graph’. The graph shows the relations (i) between the results section of the document containing the morphological description and the named graph instances (instance-based semantic anatomy graphs) that contain the instance-based semantic description graphs and (ii) between the methods section of the same document and the named graph instances (instance based semantic anatomy metadata graphs) that contain all metadata relevant to the morphological description. All semantic description named graph instances are part of an instance of the class ‘instance anatomy’, which in turn is part of the results section of the document. For reasons of clarity, resources are not represented with their URIs but with labels. *Purple-bordered box = ontology individual; purple-bordered grey box = named graph instance; yellow-bordered box with rounded corners = ontology class; arrow = property*

Above I have proposed a general structure of how to semantically represent a description document (see *Organizing a document as a semantic graph*), how to organize and semantically represent different parts of the description of the anatomical organization of a specimen in terms of named graphs of different classes (see *Morphological descriptions as Semantic Instance Anatomy Knowledge Graphs*), and how to organize and semantically represent all the metadata relevant to a particular morphological description (see *Metadata relevant to morphological descriptions*). What we still need is a scheme of how to put all this information together into a semantic description document. This can be accomplished by modeling the relation between these three layers in a named graph instance of an ‘assertions named graph’ ontology class (Fig. 8). This assertions named graph contains the ‘results section’ instance and the ‘methods section’ instance and connects them to the particular named graph instances that contain the respective data.

**Fig. 9:**
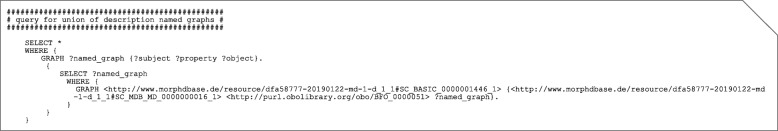
SPARQL Query for union of all named graphs that contain parts of the description. The sub-query of this query searches within the assertions named graph for all triple statements that have *Subject*: ‘instance anatomy’ and *Predicate*: ‘bfo:has part’ and retrieves a list of named graph resources (for how to query the URI of the assertions named graph and the instance of ‘instance anatomy’ see Fig. 2). The result of this sub-query lists all named graphs which contain parts of the description and thus parts of the *Anatomy Knowledge Graph*. The main query provides the union data of the list of named graphs from the sub-query (the result of this query is documented in Additional file 3)

The ‘results section’ instance has an instance of the class ‘instance anatomy’ as its part, which in turn has all the named graph instances as its part that contain instance-based semantic descriptions graphs (Fig. 8, left). The instance anatomy thus represents the *Semantic Instance Anatomy* of the specimen(s) described in this description document. The union of all the semantic graphs that are part of the instance anatomy results in the semantic morphological description recorded in the document, the *Anatomy Knowledge Graph* (see Fig. 9 for corresponding SPARQL Query; for an example file see Additional file 3).

The ‘methods section’ instance on the other hand has all the instances of ‘assay data named graph’ as its parts, each of which documents a particular observation process from which parts of the description resulted. The union of the semantic graphs that are part of the methods section results in a semantic metadata graph that documents all metadata directly referring to the description recorded in this document. Each instance of ‘assay data named graph’ specifies the parts of the description that are based on this particular observation process. The union of all the named graph instances of a document results in a semantic graph that integrates all information contained in this document (Figs. 1, 8).

Some of the ideas presented here have been implemented in a module for morphological descriptions that is currently in development for Morph·D·Base [50]. All additional files connected to this paper (see below) have been generated from SPARQL Queries using its SPARQL endpoint. The queries were conducted for a particular description entry that matches the examples given above. Morph·D·Base is a morphological data repository that is based on conventional relational database technology. It has been online since 2006. With the new description module we employ Semantic Web technology and store all data and metadata in a tuple store framework as instance-based semantic graphs, applying the general scheme described above. Users access the module through a web portal. The module’s graphical user interface enables users to generate highly standardized and formalized morphological descriptions. When describing an anatomical structure, users can reference any ontology class from any anatomy ontology that is available at BioPortal [51] and describe the structure and all of its parts as instances of these classes using HTML input forms. Each mentioned part can be further described through defined input forms, often referencing specific ontology classes from PATO [52]. Respective user input is converted to instance-based semantic graphs as described above and stored in the tuple store applying the scheme of named graphs discussed above. Since this conversion is conducted by the application, semantic graphs relating to the same underlying type of descriptive statement follow the same basic graph structure and are therefore comparable across different descriptions. All data and metadata relating to a given description can be accessed in a human-readable HTML version for browser requests and in a machine-readable RDF/OWL or JavaScript Object Notation (JSON) version for service requests. The HTML version provides the definitions for all ontology classes instantiated by described parts and properties as tooltip-texts, with links to the corresponding web resources. This substantially improves the re-usability of morphological descriptions for non-experts.

The module is currently still in development, but a prototype [53] can be accessed and functions as a proof of concept for the here proposed scheme of storing all data and metadata relating to a morphological description in a set of interlinked named graphs.

## Discussion

The general advantage of storing all data as a semantic graph in a tuple store is that it can be queried using a SPARQL endpoint, which in turn allows users to ask very detailed questions about the data in the store. Compared to relational database models, the advantage of RDF and the here proposed data scheme is that the relationships in the data are explicitly described and can directly be accessed using SPARQL. As a consequence, the conceptual model underlying the data can be fully explored using SPARQL queries. The query results, then, can be translated into various formats such as Comma-separated values (CSV) [54], and thus made ready for use in analyses tools.

The separation of information contained in a description document into several smaller semantic graphs and their organization into named graph instances of different classes allows for flexible and meaningful fragmentation of the data. A semantic description graph contained in a single named graph instance of a description document can be understood to answer a particular question based on a perceptual category with a particular described part in its focus. Each named graph class refers to an associated perceptual category. The ‘parthood named graph’ asks for direct parts of the part in focus, the ‘anatomical surface texture named graph’ for the texture quality of the part in focus, and the ‘anatomical structure volume named graph’ for the volume measured for the part in focus. The application of a network of named graph classes that are based on different perceptual categories organizes and structures the data of a description document into meaningful fragments (see also *general morphological structure concept* [2, 8, 12]) and allows for detailed metadata documentation and differentiated assignment.

Based on this structure of various named graph classes we can now flexibly define different *data views*, which can be used to meaningfully navigate the document. By specifying the classes of named graphs to be covered by a particular data view, an application can identify for any given description document or set of description documents the particular named graph instances that belong to a given view. The union of their semantic graphs represents all data contained in the document(s) that refer to that specific data view and thus all answers that the document(s) provide(s) for the questions that are associated with that particular view. A SPARQL Query template (see Figs. 2, 9) can be assigned to each data view, which enables users to query data through the application’s SPARQL endpoint without having to know SPARQL Query language.

First level views are defined based on a single named graph class, as for instance the ‘parthood named graph’ class. The union of the semantic graphs contained in all instances of ‘parthood named graph’ of a given description document will result in a semantic graph that represents all parthood relations described in that document (see Additional file 2).

Additional data views can be defined in reference to a combination of several named graph classes. The resulting views can thereby relate to one another, with one view complementing the data already covered by another view. One could, for instance, define a general-measurements view, which is defined as the union of all named graphs of a given document that contain measurement data, and a lengths-and-distances view that covers only meter-based measurements.

Fragmenting a description into individual named graphs, each of which corresponds to a single descriptive statement based on a specific perceptual category, also facilitates the alignment of different *Anatomy Knowledge Graphs*. A named graph of one description can be individually aligned with a named graph of another description in correspondence with the named graph ontology class both named graphs instantiate, knowing that named graphs that instantiate the same ontology class contain comparable information.

The fragmentability of *Anatomy Knowledge Graphs* brings about another advantage, as it allows users to search for similar fragments across various descriptions using the concept of data views discussed above. Users do not have to understand the structure of the underlying instance-based semantic graphs nor do they have to know the SPARQL Query Language in order to be able to find and re-use the information contained in descriptions, but only have to find the data view that covers the information of interest.

Unfortunately, *Semantic Phenotypes* cannot be fragmented this way, because all parts and properties mentioned in a TBox expression of a phenotype-defining ontology class are necessarily anonymous resources. When fragmenting such a class-based semantic graph into several sub-graphs, each of which is based on a single descriptive statement as discussed above, one cannot re-connect them back again to form a single connected graph. The union of these sub-graphs will not re-connect because anonymous resources that actually refer to the same particular part or property cannot be identified to refer to the same entity [41]. Another consequence of the lack of fragmentability of *Semantic Phenotypes* is that metadata statements can only be assigned to a *Semantic Phenotype* as a whole, but not to the individual descriptive statements it contains, thus failing to provide a way to differentially document the relation between each descriptive statement and its corresponding metadata. Moreover, as already mentioned above, querying the TBox expressions of *Semantic Phenotypes* using SPARQL is more complex and computationally difficult than querying ABox expressions of *Anatomy Knowledge Graphs* [39, 40].

The representation of morphological descriptions as documents that are instance-based semantic graphs consisting of various named graphs also differs from most other approaches of representing descriptions of phenotypes using ontologies. These usually involve *Semantic Phenotypes* or EQ statements that are stored in tables of relational databases, with the URIs of phenotype-defining ontology classes (or of a given E and a given Q, respectively) providing only semantic links between a specimen and its phenotype. Such solutions fail to utilize semantic technology to its full potential.

However, the actual efficiency and practical applicability of the here proposed data scheme still must be tested and compared to alternative approaches of documenting phenotypic data, such as those used in the phenoscape knowledge base [55] or in relational database models. Another issue may arise concerning the real world usability of the here proposed data scheme when implementing it in an application. In our prototype for semantic Morph·D·Base we follow the general rule to hide the actual semantic graph and data model behind HTML representations of the data. Our users do not directly interact with the data model. The interface has been developed in close collaboration with use cases led by experienced morphologists, who gave us valuable feedback regarding the usability of our approach. Users of the prototype will not necessarily need to understand the underlying structure of semantic graphs in a tuple store in order to use and benefit from this proposed semantic data model for anatomy. What still needs to be tested, though, using large amounts of data, is the scalability of the here proposed data scheme.

## Conclusion

Data contained in morphological descriptions can be very heterogeneous, ranging from purely structural specifications in the form of absent-present statements of anatomical entities to statements about their spatial properties and relations as well as their functional, developmental, and sometimes even ecological relationships. The here proposed approach, which is not limited to morphological descriptions but can be extended to any kind of phenotypic or descriptive data, allows flexible management of this data. As long as the list of underlying named graph classes is fine-grained enough, new data views can be easily defined and implemented. This is important for being able to quickly adapt to changing requirements for data exploration. When conceiving data schemes and a general structure and organization for storing morphological data and metadata in a tuple store, one cannot anticipate which aspects of the data and metadata will be of interest in future applications of the data. The here proposed use of a fine-grained scheme of named graph classes facilitates the need for a data structure that allows for efficient yet flexible data retrieval.

Moreover, the integration of morphological descriptions in a document structure has the potential to ultimately replace conventional publications of morphological descriptions in unstructured free text form. Publishing morphological descriptions as instance-based semantic graphs would make morphological data and metadata substantially more findable, accessible, interoperable, and reusable (FAIR guiding principle [7]). Morphological data would become more accessible and re-usable for non-experts, from which morphology as an academic discipline would benefit.

## Additional files

Additional file 1: document named graph. (TXT 39 kb)

Additional file 2: union of all parthood named graphs. (TXT 11 kb)

Additional file 3: union of all named graphs containing parts of the description. (TXT 18 kb)

Additional file 4: head volume named graph. (TXT 4 kb)

## Data Availability

The datasets supporting the conclusions of this article are included in Additional files 1, 2, 3 4, which contain SPARQL Queries for a given description data set in the prototype of semantic Morph·D·Base [53] together with their results.

## Abbreviations

BFO: Basic formal ontology
DCTERMS: Dublin core metadata terms
EQ: Entity-Quality
FAIR: Findable, Accessible, Interoperable, Reusable
HAO: Hymenoptera Anatomy Ontology
IAO: Information Artifact Ontology
OBI: Ontology for Biomedical Investigations
OWL: Web Ontology Language
PATO: Phenotype and Trait Ontology
RDF: Resource Description Framework
RDFS: Resource description framework schema
RO: OBO relations ontology
TI: Time interval, ontology design patterns
UBERON: Uber-anatomy ontology
UO: Units of measurement ontology
URI: Uniform Resource Identifier
XML: Extensible markup language
XSD: XML schema
